# Designing and Implementing a Metaverse Strategy for Fall Prevention in Older Adults: A Theoretical Review

**DOI:** 10.3390/jcm14207243

**Published:** 2025-10-14

**Authors:** Hongje Jang, Sangcheol Bae, John Yoo, Jongsuk Lee, Soonjang Kwon, Eunju Jung, JongEun Yim

**Affiliations:** 1 Department of Physical Therapy, The Graduate School of Sahmyook University, Seoul 01795, Republic of Korea; ghdwp325@syuin.ac.kr (H.J.); 5minlaundry@syuin.ac.kr (S.B.); gradpt@syu.ac.kr (J.Y.); liege256@syuin.ac.kr (J.L.); soonjangk@syuin.ac.kr (S.K.); hello0327@syuin.ac.kr (E.J.); 2 Department of Physical Therapy, College of Future Convergence, Sahmyook University, Seoul 01795, Republic of Korea

**Keywords:** metaverse, aging society, older adults, education program, fall prevention, virtual companion, accessibility

## Abstract

The aim of this study was to propose the use of metaverse technology as an effective educational method for fall prevention in older adults. A theoretical review was conducted by analyzing publications from PubMed/MEDLINE, EBSCO, SciELO, and Google Scholar using the search terms “metaverse,” “falls,” “older adults,” “virtual reality,” and “exercise.” From 133 identified articles published between 2000 and July 2025, and one seminal pre-2000 study included due to its foundational relevance, 52 were examined in depth. Traditional face-to-face or one-way online education often fails to meet the diverse needs and physical limitations of older adults. Metaverse-based platforms, which employ virtual avatars and immersive environments, may enhance accessibility, motivation, and social connectedness. Potential applications include personalized fall prevention training, virtual community centers, and gamified group exercise environments. Augmented and mixed reality technologies may further improve realism and usability compared with traditional virtual reality. However, challenges remain, including digital literacy gaps, device costs, and infrastructure requirements. Metaverse technology therefore offers a promising platform to bridge the gap between face-to-face and remote interventions. This review is novel in that it systematically synthesizes fragmented evidence on metaverse-based fall prevention, conceptualizes its educational potential for older adults, and provides a foundation for future clinical and policy applications.

## 1. Introduction

As the older adult population increases worldwide, Korea is on the verge of becoming a “super-aged society.” According to the 2024 Elderly Statistics, adults aged 65 and older accounted for 19.2% of Korea’s total population, and this proportion was expected to surpass 20% in 2025 [1]. However, on 23 December 2024, the population aged 65 and older had already reached 20% of the total population, and South Korea officially entered a super-aged society [2]. With the ongoing progression of population aging, recent research indicates a continuous rise in socioeconomic expenditures aimed at supporting the welfare of older adults and ensuring healthy aging [3]. Various factors hinder physical activity in older adults, including injury concerns, lack of knowledge, chronic disease, and reduced motivation or opportunity [4].

Health loss and conditions related to the aging process, such as dementia, gait disturbance, and urinary incontinence, also contribute to these limitations [5]. In addition, chronic diseases, including arthritis, cardiovascular disease, and metabolic disorders, are highly prevalent among older adults and further restrict their physical activity [6].

Each year, 26.5% of older adults worldwide experience falls, often resulting in moderate-to-severe trauma [7,8]. In Korea, the prevalence of falls among older adults was 7.2% in 2020, with a high hospitalization rate [6,9].

Older adults who have experienced more than one fall often develop a fear of falling, which increases with age and leads to greater muscle weakness, reduced mobility, and decreased life satisfaction and social activity [10]. Those with chronic conditions such as stroke, cancer, arthritis, depression, and diabetes are also at higher risk of falls compared with healthier peers [11]. Moreover, the number of chronic diseases in adults over 65 is directly related to fall recurrence, further limiting physical activity and worsening age-related diseases [12].

Therefore, innovative platforms such as the metaverse, which provide interactive and safe environments, could effectively address these barriers and improve participation in fall prevention programs. However, despite growing interest in digital and virtual reality interventions, no comprehensive review has systematically synthesized the role of metaverse-based approaches in fall prevention for older adults. This gap highlights the need for the present theoretical review. Accordingly, this study aims to explore and propose a new paradigm for fall prevention exercise programs for older adults by reviewing the potential application of metaverse technology. Specifically, we examine how metaverse-based interventions can overcome the limitations of existing face-to-face and non-face-to-face programs and improve accessibility, engagement, and effectiveness in fall prevention for older adults.

## 2. Methods

A theoretical review was carried out as part of a research project to determine the current state of knowledge on the subject under study. The review encompassed publications on the metaverse, including trends, problems of the current situation, definitions, advantages, applications, and future strategies, published in the period from 2000 to July 2025. Database searches of PubMed/MEDLINE, EBSCO, SciELO, and Google Scholar, considering clinical cases, review articles, and clinical trials using the search terms “metaverse”, “falls”, “older adults”, “virtual reality”, and “exercise”, and identified 133 records. In addition, one seminal pre-2000 study was included because it was among the earliest large-scale investigations to examine fear of falling in community-dwelling older adults. Although published before the main search period, this study remains a landmark reference in the field, as it conceptualized fear of falling as a measurable barrier to physical activity and continues to inform contemporary fall-prevention strategies, including the rationale for technology-assisted interventions.

The inclusion criteria were broadly defined to encompass peer-reviewed publications related to aging, health promotion, falls, exercise, or physical activity, particularly those involving digital, immersive, or technology-assisted interventions (e.g., metaverse platforms, virtual reality, augmented reality, gamification, wearable or robotic devices, ICT-based programs, and online or remote health services). Populations could include older adults or mixed-age groups, provided that the findings offered conceptual or practical implications for healthy aging or fall prevention. The exclusion criteria included duplicate records, purely technical or marketing papers without health applications, non-peer-reviewed sources, and publications not written in English or Korean.

**Figure 1:**
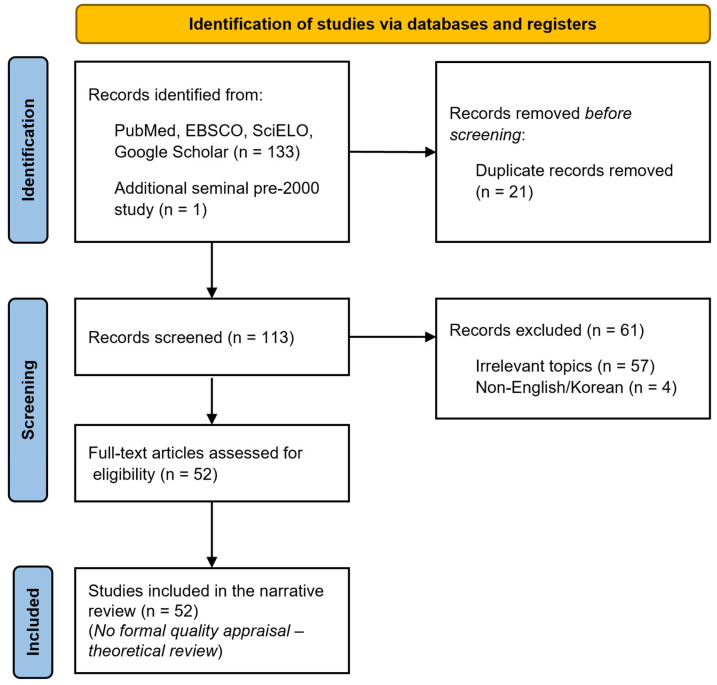
Flowchart of the study selection process. Records were identified from databases for 2000–July 2025, and one pre-2000 seminal study was additionally considered. Duplicates were removed, titles/abstracts screened, and eligible full texts included (n = 52). No formal quality appraisal was undertaken given the theoretical scope.

After duplicate removal and screening, 52 studies were analyzed in depth (Figure 1). Because this study was conducted as a theoretical review, no formal quality assessment of the included studies was performed.

## 3. Main Theory

### 3.1. Trends in Fall Prevention Programs for Older Adults

Surveys and reviews consistently point to supervised, group-based formats as widely implemented and effective components of older-adult fall-prevention programs [13,14,15,16]. Studies have shown that approximately 40% of older adults participate in institutional settings to improve their physical strength and health and that users of senior welfare centers commonly report a need for exercise programs [13]. Welfare service institutions provide various healthcare services to meet the needs of older adults. According to a 2018 survey, 222 out of 304 senior welfare centers in Korea (73.0%) implement “functional recovery programs” that provide services such as physical therapy and exercise rehabilitation, and 269 (88.5%) implement “health promotion programs” that provide services such as health education and counseling [14]. According to a domestic meta-analysis of exercise programs for health in older adults, full-body strength enhancement, dance sports, lower extremity band exercise, senior aerobics, walking, balance training, step-by-step resistance exercise, and complex fall prevention programs are highly effective in preventing falls [15]. These interventions were mostly conducted in leisure welfare facilities, where experts provided programs that included approximately 60 min of group exercise three times a week [16]. Therefore, group education in these facilities appears to leverage the “companion effect.”

Companion exercise (involving sports and leisure activities) is a physical activity performed by several people together and a social activity that requires punctuality, maintenance of order, implementation of rules, and courtesy. Those who participate in companion exercise interact with each other and form interpersonal relationships with various classes, and sociality and morality are cultivated through relationships with others. The physical activity in companion exercise does not refer only to competitive sports performed according to rules. However, this concept collectively relates to leisure activities, including all physical activities that promote physical, social, moral, and mental health [17]. In a study on companion exercise among older adults, No et al. found physical activity among older adults was higher at every level when exercising with companions compared with when they exercised alone, and those companions have a protective effect on maintaining healthy lifestyles in this age group. Thus, beyond improving health, the influence of companion exercise also plays a role in controlling behaviors that adversely affect health by having a positive effect on physical activity levels and health maintenance in older adults [17].

### 3.2. Problems of the Current Situation

Older adults typically have physical limitations, such as declining cognitive abilities and limited mobility, and can be passive in acquiring health information, making it difficult for them to continue participation in face-to-face education [18,19]. Furthermore, according to statistics released by the Ministry of Health and Welfare, less than half of older adults receive care services compared to vulnerable older adults living alone [20]. Therefore, educational or exercise programs for older adults require customized learning tools.

Multiple physical, psychological, and socio-environmental barriers interact to restrict physical activity participation among older adults. Specifically, physical limitations—including chronic diseases, reduced mobility, pain, and fear of falling—psychological factors such as lack of motivation, diminished self-efficacy, and depression or anxiety, as well as environmental issues such as inadequate social support, limited accessibility, and safety concerns, have all been identified as major impediments in this population [21].

Although these barriers have long impeded older adults’ participation in physical activity, recent changes in the external environment have further exacerbated the situation. In particular, the environment for exercise participation among older adults has further deteriorated as a result of the COVID-19 pandemic and other large-scale infectious disease outbreaks. During the pandemic, social distancing guidelines, long-term closures of public gyms and welfare facilities, restrictions on face-to-face gatherings and activities, and psychological anxiety about infection all contributed to this deterioration. As a result, not only was the use of welfare facilities for the older adults, such as senior citizen centers, suspended, but interaction and communication with others and various physical activities in small spaces were also greatly reduced [22].

To address these changes, educational institutions for older adults are attempting to introduce online content that can enable education and communication in non-face-to-face situations as well as beginning to communicate with seniors who are capable of online education using text messages, social media, and facility websites. In addition, they provided non-face-to-face education by piloting home training, hobby, and leisure programs through YouTube and operating real-time communication channels through Zoom [23]. Thus, welfare centers have moved their service delivery methods online to minimize service gaps. A survey conducted in September 2020 of 60 welfare centers across Korea found that 24 provided videos demonstrating basic movements for health promotion and recovery. The introduction and expansion of non-face-to-face programs have facilitated increased participation of older adults in online home-based training and remote leisure activities.

However, simply introducing these programs does not address all the problems, and it is necessary to assess how these changes have impacted the physical activity and quality of life of older adults in practice. Meanwhile, surveys on satisfaction with program delivery methods found that in-person exercise programs were more satisfying for older adults than non-face-to-face programs [24]. The primary reasons for higher satisfaction with in-person programs included the ease of communication, the presence of highly skilled role models, and the emotional support provided by professional trainers with expertise [17]. However, dissatisfaction with in-person programs was also reported, mainly due to the burden of socializing, time and physical constraints of real-life companions, and difficulties in finding appropriate partners for exercise [22].

Non-face-to-face exercise programs offered advantages such as repeatability, freedom from the gaze of others, and flexibility in time and location. However, the most common cause of dissatisfaction was the lack of communication, as existing non-face-to-face programs often relied on one-way information delivery, did not adequately reflect individual characteristics, and were mainly delivered through video viewing. Interestingly, studies found that when a rehabilitation device enabling remote physician monitoring was used, satisfaction with remote rehabilitation (78.3%) was more than double that of in-person rehabilitation (36.7%) [24]. These findings suggest that even in non-face-to-face settings, higher satisfaction than current in-person programs can be achieved if active communication and tailored support are provided. Given these findings, it becomes clear that more advanced technological solutions are necessary to bridge the gap between traditional and digital service delivery.

On the other hand, even after the pandemic subsided and in-person services at welfare centers resumed, the participation rate of older adults in these programs has not fully recovered to pre-pandemic levels. This appears to be due to lingering structural barriers such as habits developed during prolonged non-face-to-face living, ongoing concerns about health and infection, and reduced mobility. In addition, many welfare centers and related institutions face practical difficulties such as budget cuts, staff shortages, and reduced operating hours [25]. These combined factors have further delayed the revitalization of in-person exercise programs for older adults.

### 3.3. Definition, Advantages, and Applications of the Metaverse

These considerations set the stage for outlining the metaverse—its definition, advantages, and applications. The metaverse is being recognized as a next-generation space that crosses the boundary between reality and virtuality. Metaverses consist of augmented reality, life-logging, and mirror and virtual worlds, allowing users to realize anything and imagine in any form [23]. These metaverses include virtual reality, which is lifelike; augmented reality, which superimposes virtual reality on natural space; mixed reality, which combines the above two technologies; and extended reality, which is a realistic representation of the world that makes it difficult to distinguish between what is virtual and what is real [26].

Research is underway on information and communication technology (ICT)-based senior education platforms such as “digital sarangbang” to build a metaverse-based senior education system. For example, Gyeonggi-do’s City B has been operating a comprehensive ICT welfare center for older adults since the second half of 2020, providing dementia prevention and physical health programs using AI robots. Older adults can use various ICT devices, receive digital education, and wear devices intended for personalized health management [27]. Furthermore, as research on metaverse-based digital literacy education continues, new attempts are being made to incorporate metaverse technology into educational programs for older adults [23].

Metaverse technology not only extends the real world but also improves the constraints older adults face, such as accessibility issues and device usage restrictions, and facilitates interactions between users to achieve a companion effect. Nevertheless, the high costs associated with some metaverse-related devices and required infrastructure may limit immediate widespread adoption among older adults. Within this context, the applications and limitations of virtual and augmented reality warrant examination.

#### 3.3.1. Virtual Reality and Augmented Reality: Applications and Limitations

Studies using virtual reality are being conducted as next-generation educational programs for older adults to prevent decline in physical function caused by age-related diseases and improve outcomes. These studies have reported improved strength, balance, walking ability, static balance control, and fall efficacy in older adults, and educational virtual reality devices for families of patients with dementia are also being developed [28,29,30]. Research on fall prevention programs using virtual reality is also underway, showing the impact of virtual reality on the risk of falls in older adults and confirming that virtual reality interventions can increase static balance and fall efficacy and reduce fall risk [15]. Efforts have been made to overcome the practical limitations of programs for older adults by utilizing virtual reality technology, such as developing virtual reality content to measure physical function in older adults [31].

However, in virtual reality, when inconsistencies exist between visual and vestibular sensory information in a virtual environment, conflicts between these two senses are likely to occur, resulting in symptoms such as dizziness, vomiting, nausea, and headache. In addition, virtual reality requires a head-mounted display, which is an expensive piece of equipment that must be worn on the face, making it inconvenient for older adults [32]. Senior Welfare Center A in Gyeonggi-do, a smart healthcare center, operates to improve users’ physical activity and cognitive functions and resolve digital literacy through an augmented reality physical and cognitive activity program [33]. Chen et al. stated that augmented reality-based programs to reduce fall risk could provide a higher level of realism than virtual reality-based programs. Subsequently, research findings have shown that education utilizing augmented reality technology provides higher satisfaction than virtual reality and is good in practical use. This demonstrates that augmented reality technology is a more suitable educational method for older adults than virtual reality [34]. Despite augmented reality’s practical advantages over virtual reality, residual device burden and social and motivational gaps warrant examination of metaverse-based social virtual environments. A side-by-side summary of companion exercise, virtual reality, and augmented reality—aligning reported benefits with delivery constraints—is provided in Table 1.

#### 3.3.2. Transition to Metaverse and Social Virtual Environments

Nevertheless, in parallel with these advances in augmented reality, recent studies have also attempted to address the limitations of conventional virtual reality by introducing metaverse-based, social virtual reality environments. While these platforms still face the physical constraints of virtual reality equipment, they seek to offset some of the social and motivational barriers of exercise for older adults by enabling real-time avatar-based interaction and companionship. For example, Shah et al. (2022) found that older adults participating in avatar-mediated social virtual reality exercise programs reported increased engagement and motivation, suggesting that the social and emotional benefits of in-person group exercise can be partially replicated in a virtual environment, despite the remaining physical discomforts of virtual reality [35].

Given that both in-person and non-face-to-face programs have inherent strengths and limitations, and that full recovery of participation has not yet been achieved in the post-pandemic era, recent efforts have focused on supplementing these issues through the adoption of advanced technologies such as virtual reality and virtual companion-based programs [17]. Virtual companions can work out anywhere and anytime and can be customized to the user’s desired intensity. This can benefit athletes who have difficulty finding a companion because of their high fitness level, rehabilitation patients who have difficulty exercising because of their low fitness level, people marginalized from the movement due to lack of places and experts, and older adults. However, most cases also have limitations, such as the lack of immersion and realism created by virtual reality exercises provided through a screen. Furthermore, users view virtual companions as unrealistic [36]. This shows that online programs can only partially replace traditional face-to-face training and that virtual companionship exercises are not suitable for all situations.

Therefore, new training platforms need to be developed to overcome the “lack of communication” and maintain the “companionship effect” even in non-face-to-face situations. Such a platform would enable user interaction and provide new training experiences. One promising approach to overcome these limitations is the adoption of metaverse technology, which has recently received increasing attention in the field of senior health and education. In this context, there has been a reported case where an avatar-based metaverse exercise platform was specifically developed and applied for older adults. For example, Hong et al. developed and implemented a “fashion model walking” exercise intervention for older adults, utilizing a Kinect sensor and 3D avatars to deliver real-time feedback. Participants were able to view their movements via avatars and received immediate postural and gait-related feedback during the sessions. The intervention led to significant improvements in balance, physical coordination, and overall engagement and satisfaction. These findings suggest that avatar-mediated real-time feedback may be particularly effective in enhancing exercise motivation and posture correction in older adults [37].

#### 3.3.3. Device-Light Mixed Reality and Avatar-Mediated Platforms

To address equipment burden while preserving social interaction and feedback, device-light mixed-reality and avatar-mediated platforms are being explored. “Real Cube” (KT Corporation, Seoul, Republic of Korea) enables mixed reality without using head-mounted displays or augmented-reality glasses by linking a beam projector with a wall-mounted recognition sensor and a motion-recognition sensor in real space. When the user moves in line with the screen implemented using a beam projector to perform an activity that incorporates sports, the sensor detects that movement and reflects it in the results, providing educational screen sports that benefit users’ cognitive and physical development [38]. This technology has been previously used in dementia prevention physical education activities conducted at senior-related organizations, such as K Senior Plaza [39].

Metaverse platforms also allow users to have direct or indirect experiences and interactions through characters. For example, Lim et al. (2022) found that learning environments utilizing avatars can increase overall interest levels, with social interaction between learners playing a positive role [40]. Thus, Microsoft launched Mesh, a mixed-reality-based remote collaboration platform that allows users to view real-world objects and virtual information simultaneously, enabling remote collaboration in various fields such as healthcare, manufacturing, and design. In addition, other metaverse-based collaboration platforms such as “Spatial” and “Glue” are being developed [41]. In Korea, ifland and ZEPETO have the advantage of overcoming physical disabilities and enabling interactions between users within the metaverse by using 3D characters to allow activities without space and time constraints.

Notably, a study involving older adults using a prototype virtual social welfare center demonstrated that avatar-based, real-time interactions significantly improved emotional stability and social connectedness. Participants initially gathered to communicate but gradually formed relationships based on shared interests, which had a positive long-term effect on their psychological well-being compared to non-participants [42]. Therefore, allowing multiple participants to enter a virtual situation, express themselves through avatars, and interact with other participants may maximize the effectiveness of companion exercises, thereby generating emotional interactions and interest among users and potentially motivating physical activity among older adults [17,23,43].

Metaverses can also provide older adults with environments that stimulate positive memories and imagination. Using virtual reality to materialize the process of recalling positive memories may be a valid methodology to promote behavioral activation [44]. Participants who experience biophilic environments in virtual worlds were found to have physiological and cognitive responses similar to those during real-world encounters, and virtual spaces in outdoor environments can induce positive emotions [45,46]. These findings suggest that metaverse-based exercise programs may be a promising tool for enhancing positive mood and emotion regulation strategies.

Thus, tapping into childhood memories or imagined environments could provide an emotional connection to exercise, increasing one’s motivation for an exercise program. For example, exercising in a virtual environment that mimics one’s favorite childhood playground or park could stimulate positive emotions and memories, making the exercise more enjoyable and motivating [47]. Thus, the use of virtual avatars within the metaverse to compensate for physical disabilities and environmental factors that must be overcome for older adults to participate in wellness programs can be extended to spaces within the metaverse to increase accessibility, making it a more inclusive learning tool [48].

In addition, wearing a small device that measures participants’ exercise status that program providers can monitor would allow older adults to take the initiative to participate in the program while providing customized training and exercise programs based on their health status, learning speed, and space constraints, as well as individualized services based on data analysis.

### 3.4. Future Strategies for Using the Metaverse in Fall Prevention Programs for Older Adults

**Figure 2:**
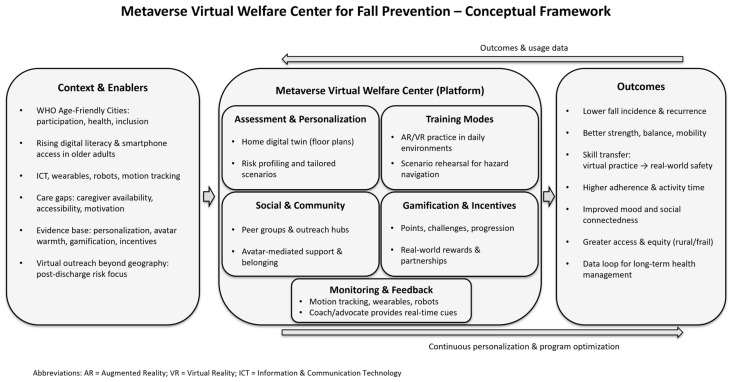
Conceptual framework of a Metaverse Virtual Welfare Center for fall prevention in older adults.

The World Health Organization defines Age-Friendly Cities as places where people do not feel uncomfortable becoming older, people of all ages can live, and older adults can actively participate in their communities to live vibrant and healthy lives [49]. Therefore, we propose applying the metaverse as a platform for the online delivery of in-person services. Metaverse platforms have been utilized in various fields, such as gaming, daily life, and industry. There is a growing view that people will spend more time in the metaverse in the future and that the transition will accelerate as more economic and social activities in the real world are connected to or converge with the virtual world [41]. Accordingly, virtual administrative agencies can be created in metaverse spaces. Civil complaints can be handled using avatars, and virtual libraries can be constructed to provide users with immersive and new reading experiences. In addition, the metaverse platform can be utilized to respond to social issues such as the gap in non-face-to-face education and marginalization of rural areas due to geographical disparities [41] (Figure 2).

#### 3.4.1. Program Model and Personalized/Socialized Fall Prevention

The metaverse can serve as a safe and effective platform for personalized fall prevention training by providing a customized virtual environment that faithfully recreates an individual’s living space and lifestyle. Users can practice fall prevention techniques in an augmented reality environment superimposed on top of their living space, making it easier to apply these techniques in real life. This also allows older users to connect with peers in the metaverse who share similar experiences, fostering a sense of community and belonging. In terms of fostering emotional connections, research has shown that avatar-based interactions provide warmer and more supportive social experiences compared to text-based platforms. This highlights the importance of designing metaverse environments that enable emotional support and social bonding, particularly for older adults [50].

While traditional outreach programs for older adults take place in local communities, the metaverse allows people to come together in virtual outreach centers based on hobbies, interests, or shared experiences rather than geographic location [51]. Older patients are prone to experiencing falls after hospital discharge. Research has shown that individualized components and expanded prevention programs can reduce the incidence of falls; thus, by envisioning different real-world scenarios based on individual needs and circumstances within the metaverse, older adults can practice fall prevention skills in a safe and controlled environment before applying them in their daily lives [52,53]. In an effectiveness study on the incidence of falls among discharged orthopedic patients, Ueda et al. (2022) found that, compared to a control group that received only standard care, the group that received a customized fall prevention program using home floor plans in addition to usual care showed a greater reduction in the incidence of falls [54]. Based on these findings, metaverses can be used to create digital environments tailored to individuals’ specific needs, allowing older adults to connect with others with similar interests to make their exercise routines more exciting and enjoyable. By participating in fall prevention programs, seniors will be able to build meaningful relationships with others and improve their mental health and overall quality of life.

A virtual welfare center can be created in the metaverse space that users access by creating avatars using an optical motion-tracking camera. Wearable devices or robots used to synchronize the appearance and movement of the user and the avatar can increase user embodiment [55,56]. Through their avatars, users can participate in educational and exercise programs in a virtual wellness center at their convenience. In particular, in exercise programs, users can see their companions participating simultaneously and increase the time spent and engagement in physical activity, which may positively affect physical activity levels and health maintenance among older adults [17]. Users will be able to see their companions exercising with them beyond what can be shown on a traditional screen, which is likely to improve engagement and realism compared to previous exercise programs with “virtual companions.” A virtual wellness advocate (e.g., a service provider or expert) can provide real-time personalized feedback and monitoring based on information collected through a device worn by the user. The direct and indirect interactions between users are expected to improve psychological functioning from a social support perspective and reduce stress [17].

Under new standards, healthcare for older adults must address three issues: lack of helpers, accessibility, and motivation [57]. The Metaverse Virtual Welfare Center is one way to solve these problems. The Metaverse Virtual Welfare Center promotes older adults’ use of the metaverse, thereby reducing the digital divide and improving their quality of life and satisfaction. This will increase older adults’ social self-efficacy by naturally inducing disconnected social activities. Participating in a fall prevention exercise program with real-world companions in a virtual welfare center located within the metaverse is predicted to not only prevent falls in older adults but also positively affect the incidence and prevalence of falls in this population over time. This will promote the health of older adults and make it possible to accumulate the data necessary for future health management, which can be utilized to help older adults manage their health. Thus, the Metaverse Virtual Welfare Center can serve as a health guide for older adults.

#### 3.4.2. Engagement Mechanisms: Gamification and Incentives

Furthermore, with the proper use of gamification and incentives, older adults’ motivation and social interactions while participating in fall prevention programs can potentially be increased. Gamification, the application of game design elements such as points and challenges to non-game contexts, improves attitudes toward and enjoyment of exercise and increases the time individuals spend engaged in physical activity [58]. Combining game design elements can make gamification more effective, thus increasing the psychological impact on participants [59]. In a recent study in Korea, a metaverse-based indoor cycling program incorporating gamification for older adults with mild cognitive impairment showed improvements in both physical function and engagement, supporting its potential in fall prevention interventions [60].

Incentives are also known to be effective in improving exercise behavior, with financial incentives, in particular, being shown to increase exercise session attendance in six months or less, thereby increasing exercise persistence in adults [61,62]. Therefore, designing a fall prevention program as a game with specific goals could increase user engagement and motivation. Offering real-world incentives for achieving these goals, such as fitness equipment, discounts on services, or partnerships with communities that offer services or promotions, could increase participation in fall prevention programs. Furthermore, providing opportunities to give physical gifts to friends and family based on achievements could encourage social interaction from the metaverse to the real world.

#### 3.4.3. Digital Readiness, Implementation, and Reporting

Digital literacy is also a crucial factor to consider when applying digital technology-based education and services for older adults. As of 2020, the digital literacy level of older adults in Korea was 68.6% that of the general population, which is lower than that of people with disabilities (81.3%) and farmers and fishers (77.3%). However, between 2017 and 2020, the overall digital literacy level among older adults increased from 58.3% to 68.6%, access to digital literacy from 89.9% to 92.8%, digital literacy competency from 41% to 53.7%, and digital literacy utilization from 59.9% to 71.4%. Smartphone penetration is also increasing (76.6%), facilitating an increase in Internet access among older households to 85.3% and the “use of search, email, and content services” among older adults increasing to 81.5% [23]. These trends suggest that various forms of digital media are available to educate older adults. ICT-based programs, which have gained attention as healthcare, home care, and communication tools, are currently being applied in continuing education for older adults, contributing to various activities that improve their overall quality of life.

ICT-based programs and wearable devices are expected to provide continuous connectivity between subjects and objects and real-time remote support services via mobile devices in education for older adults [23]. Overseas, avatar-mediated educational programs for older adults have already been implemented in virtual settings, and metaverses that support leisure activities customized for older adults have been developed in Korea [23,63]. Older adults who participated in the domestic program were highly satisfied with the outcome; in particular, they wanted to receive education that could lead to employment by acquiring appropriate certifications as well as education that involved relevant physical activity. This suggests that demand will emerge for physical activity in metaverse welfare centers, which will promote older adults’ motivation to participate in society [63].

Finally, to ensure feasibility and equity in low-resource settings and in low- and middle-income countries, prioritize device-light, hub-based delivery (smartphone or tablet with a camera or inertial sensors) consistent with the World Health Organization’s digital-health guidance on acceptability, feasibility, resource use, and equity [64]. Programs should include simple triage and routine tracking of minimum implementation indicators—exposure volume, supervision level, adverse events, reasons for dropout, and basic per-participant costs—drawing on the implementation outcomes and the RE-AIM (Reach, Effectiveness, Adoption, Implementation, and Maintenance) framework [65]. Report digital components using the mERA (mobile health Evidence Reporting and Assessment) checklist, and support scale-up by training community health workers and establishing privacy-compliant referral and monitoring pathways [66].

### 3.5. Strengths and Limitations

This theoretical review’s strength lies in integrating diverse studies into a single framework to present practical decision criteria (target population, setting, and resource conditions). As a theoretical review, however, we did not conduct a formal quality appraisal. Limitations include heterogeneity in study designs, sample sizes, and supervision levels; short follow-up periods; insufficient reporting of safety and reasons for dropout; a paucity of evidence on cost and equity; and inconsistent definitions of terms. Consequently, future studies should compare lightweight implementations (smartphone- or tablet-based web sessions with camera- or inertial-sensor motion tracking) with head-mounted-display–centered interventions using randomized or stepped-wedge designs. They should report both implementation indicators (acceptability, feasibility, fidelity, and reach) and cost-effectiveness, while standardizing minimum reporting items—exposure dose (frequency, duration, and intensity), co-presence signal (synchronous participation), supervision level, safety screening/adverse-event reporting, and reasons for attrition. In practice, metaverse-enabled delivery can provide interactive tasks and immediate feedback using smartphones or tablets and camera/inertial sensors without requiring expensive dedicated hardware. Equipment can be pooled and circulated through local hubs such as senior welfare centers, public health centers, and community-based organizations. A facilitation model in which community health workers handle onboarding, safety checks, equipment sharing, and schedule coordination is recommended. Pairing standardized safety screening with brief weekly synchronous participation sessions can enhance feasibility, safety, and equity even in low-resource settings and help reduce regional disparities in access.

## 4. Conclusions

This study reviewed domestic and international research on non-face-to-face educational programs for older adults, including online and virtual reality platforms, aiming to address service gaps and improve accessibility for the aging population.

First, domestic studies indicate a need for more interactive approaches beyond traditional video-based online content. Incorporating metaverse technology offers a promising strategy to enhance engagement and effectiveness. However, digital literacy gaps among older adults remain a significant barrier, necessitating targeted interventions to improve technological accessibility.

Second, international evidence supports avatar-based educational interventions, with remote rehabilitation devices providing real-time feedback showing higher satisfaction than traditional face-to-face methods. Despite initial success, sustaining long-term participation remains a challenge that must be addressed.

Third, establishing a metaverse-based virtual welfare center could serve as an innovative platform for delivering comprehensive health and leisure programs to older adults, especially in non-face-to-face contexts. Nonetheless, attention must be paid to financial and practical accessibility issues related to technology implementation.

Future research should continue to explore these effectiveness, accessibility, and digital literacy challenges. Strengthening collaboration among healthcare, technology, and policy sectors will be essential to fully realize the potential of metaverse technologies for promoting healthy aging.

## Figures and Tables

**Table 1:** Comparative summary of companion exercise, virtual reality, and augmented reality for older adults’ fall prevention delivery.

Modality	Pros (from Current Text)	Cons (from Current Text)
Companion Exercise	Social activity fostering interpersonal relationships and sociality/morality (Section 3.1); higher activity at every level with companions and protective effect on healthy lifestyle [17].	Dissatisfaction due to the burden of socializing; time/physical constraints of real companions; difficulty finding appropriate partners [22].
Virtual Reality	Improved strength, balance, walking ability, static balance control, and fall efficacy in older adults [28,29,30]; reduced fall risk [15]; development of content to measure physical function in older adults [31].	Visual–vestibular conflict → dizziness, nausea, vomiting, headache; head-mounted display is expensive and inconvenient for older adults [32].
Augmented Reality	Augmented reality physical/cognitive activity program [33]; higher realism and satisfaction than virtual reality [34]; practical usability; may be more suitable for older adults than virtual reality [34].	

## Data Availability

The data presented in this study are available upon request from the corresponding author.

## References

[1] Statistics Korea 2024 Elderly Statistics. https://www.kostat.go.kr/board.es?act=view&bid=10820&list_no=432917&mid=a10301010000.

[2] Ministry of the Interior and Safety The Proportion of the Population Aged 65 or Older Exceeds 20%. https://www.mois.go.kr/frt/bbs/type010/commonSelectBoardArticle.do?bbsId=BBSMSTR_000000000008&nttId=114622.

[3] Kallestrup-Lamb M., Marin A.O., Menon S., Søgaard J. (2024). Aging populations and expenditures on health. J. Econ. Ageing.

[4] Seong C.H., Yoo L.K., Jang C.O. (2008). Conceptual structure of exercise constraints and the difference according to the stage of change in the aged. Korean J. Sport Psychol..

[5] Kim C. (2006). Characteristics of geriatric diseases. Korean J. Med..

[6] Ministry of Health and Welfare (2021). A New Generation of Seniors is Emerging, and It’s a Different Generation-2020 Senior Living Survey Results Released. https://www.directsupply.com/wp-content/uploads/2020/12/2020-Senior-Housing-Survey-Whitepaper_single-pages_150dpi.pdf.

[7] Salari N., Darvishi N., Ahmadipanah M., Shohaimi S., Mohammadi M. (2022). Global prevalence of falls in the older adults: A comprehensive systematic review and meta-analysis. J. Orthop. Surg. Res..

[8] World Health Organization Falls. https://www.who.int/news-room/fact-sheets/detail/falls.

[9] Korea Disease Control and Prevention Agency (KDCA) (2022). Korea National Hospital Discharge In-Depth Injury Survey, 2004–2019.

[10] Arfken C.L., Lach H.W., Birge S.J., Miller J.P. (1994). The prevalence and correlates of fear of falling in elderly persons living in the community. Am. J. Public Health.

[11] Paliwal Y., Slattum P.W., Ratliff S.M. (2017). Chronic health conditions as a risk factor for falls among the community-dwelling US older adults: A zero-inflated regression modeling approach. BioMed Res. Int..

[12] Immonen M., Haapea M., Similä H., Enwald H., Keränen N., Kangas M., Jämsä T., Korpelainen R. (2020). Association between chronic diseases and falls among a sample of older people in Finland. BMC Geriatr..

[13] Chong H.-C., Sohn T.-Y. (2019). Health Status and Health Care Service Program Needs of Senior Welfare Center Users. Korean J. Health Serv. Manag..

[14] Choi J.-Y., Park S.-J. (2019). An Analysis of Education Space Program in Senior Citizens’ Welfare Center by Elderly Learning Types. J. Archit. Inst. Korea.

[15] Lim K., Choi W., Park H.-E., Lee J.-E., Park S.-Y. (2019). Understanding and Prevention of Fall-related Injuries in Older Adults in South Korea: A Systematic Review. Phys. Ther. Korea.

[16] Park J.H., Kim H.-J. (2022). Characteristics and effects of fall prevention interventions among the Korean older adults: A systematic review. J. Korean Gerontol. Nurs..

[17] No E.J., Lee M.-C., Han K. (2021). Research trend of the exercise companion and future direction-Focusing on Metaverse & Virtual Reality. J. Korean Leis. Sci..

[18] Lee S.H., Lim C.H., Kim W.C. (2020). An Exploratory Study on the Possibility of Using Next-Generation Technology in Long-term Care Facilities: Focusing on the Perception of the Workforce of in Long-term Care Facilities. J. Korea Acad. Ind. Coop. Soc..

[19] Na K., Jeong Y. (2017). Exploring Older Adults’ Experienced Barriers and Emotional Changes in Seeking Health Information. J. Korean Libr. Inf. Sci. Soc..

[20] Lee G.E., Lee H.G., Cho N.H., Lee J.-H. Mobile Platform to Prevent Depression in the Elderly Using Smart Home Garden. Proceedings of the Annual Conference of KIPS 2022.

[21] Mbabazi J., Kanmodi K.K., Kunonga E., Tolchard B., Nnyanzi L.A. (2023). Barriers and facilitators of physical activity. J. Health Allied Sci. NU.

[22] Oh-Jung K. (2020). A Case Study of Changes in the Exercise Behavior of the Elderly by COVID-19. Korean J. Sport Psychol..

[23] Lee H.-S., Kim H.C. (2022). Metaverse and the Future of education for the elderly: Focusing on the Liberal Arts Education for the Elderly in Senior Welfare Centers. Korean J. Educ. Gerontol..

[24] Lee D.-S., Lee H.-S., Kim R.-H., Kim Y.-S., Kim C.-Y., Park B.-S., Park B.-H., Shin M.-J., Oh E.-J., Woo S.-M. (2023). Effects of Face-to-face and Non-face-to-face Exercise Programs on Exercise Satisfaction for the Elderly in the Community. Korean Soc. Phys. Med..

[25] Armitage R., Nellums L.B. (2020). COVID-19 and the consequences of isolating the elderly. Lancet Public Health.

[26] Yang J.O., Lee J.S. (2021). Utilization exercise rehabilitation using metaverse (VR· AR· MR· XR). Korean J. Appl. Biomech..

[27] Choi H.-S. (2020). Direction of elderly welfare in the corona era. Public Policy Mon..

[28] Song C., Shin W.-S., Lee K.-J., Lee S. (2009). The Effect of a Virtual Reality-based Exercise Program Using a Video Game on the Muscle Strength, Balance and Gait Abilities in the Elderly. J. Korea Gerontol. Soc..

[29] Kim E.J., Hwang B.Y., Kim M.S. (2010). The Effect of a Virtual Reality Program on Static Balance Control and Fall Efficacy of Elderly People. J. Korean Gerontol. Soc..

[30] Cheon H., Jung S., Kim J., Yang Y., Kim G.J., Song J.-A. (2022). Characteristics and Preferences Related to Using Virtual Reality Devices for Education of Families of Persons with Dementia: A Descriptive Study. J. Korean Gerontol. Nurs..

[31] An Y., Lee W., Bae S., Cho J. Development of SPPB Virtual Reality Contents for the Elderly. Proceedings of the HCI Korea 2019.

[32] Lee C., Lee J., Jwa M., Jung J.S., Baek J., Kim H.D. (2022). Based on Augmented Reality Development and Evaluation of Cognitive Training Applications for Elderly. J. Occup. Ther. Aged Dement..

[33] Bundang Senior Welfare Center Digital-Based Welfare Smart Healthcare Center. https://web.archive.org/web/20231128140150/https://bdsenior.or.kr/page/VP21120207799046?menu=215&menuitem=1218.

[34] Chen M., Tang Q., Xu S., Leng P., Pan Z. (2020). Design and evaluation of an augmented reality-based exergame system to reduce fall risk in the elderly. Int. J. Environ. Res. Public Health.

[35] Shah S.H.H., Hameed I.A., Karlsen A.S.T., Solberg M. Towards a social vr-based exergame for elderly users: An exploratory study of acceptance, experiences and design principles. Proceedings of the International Conference on Human-Computer Interaction.

[36] Anderson-Hanley C., Snyder A.L., Nimon J.P., Arciero P.J. (2011). Social facilitation in virtual reality-enhanced exercise: Competitiveness moderates exercise effort of older adults. Clin. Interv. Aging.

[37] Hong S., Park S., Jung S. The Effect of Fashion Model Walking Program Using Kinect on the Movement Activity of the Elderly. Proceedings of the HCI International 2023-Late Breaking Papers.

[38] KT Enterprise Real Cube. https://web.archive.org/web/20230522170844/https://enterprise.kt.com/pd/P_PD_BS_VS_001.do#kt_pc_%EC%84%9C%EB%B9%84%EC%8A%A4%20%EC%86%8C%EA%B0%9C.

[39] Lee J.-W. KT Supports Senior Dementia Prevention Activities with Metaverse ‘RealCube’. http://archive.today/2025.08.12-035152/https://www.apnews.kr/news/articleView.html?idxno=1833995.

[40] Lim T., Ryu J., Jeong Y. (2022). The Effects of Emotional Interaction by Avatar on Presence and Interest Development in the Metaverse Learning Environment. Korea Educ. Rev..

[41] Han S. (2021). The state and future of the Metaverse platform. FUTURE HORIZ..

[42] Liang H., Li J., Wang Y., Pan J., Zhang Y., Dong X. (2023). Metaverse virtual social center for elderly communication in time of social distancing. Virtual Real. Intell. Hardw..

[43] Stokel-Walker C. (2022). Welcome to the metaverse. New Sci..

[44] Fernandez-Alvarez J., Colombo D., Suso-Ribera C., Chirico A., Serino S., Di Lernia D., Palacios A.G., Riva G., Botella C. (2021). Using virtual reality to target positive autobiographical memory in individuals with moderate-to-moderately severe depressive symptoms: A single case experimental design. Internet Interv..

[45] Yin J., Zhu S., MacNaughton P., Allen J.G., Spengler J.D. (2018). Physiological and cognitive performance of exposure to biophilic indoor environment. Build. Environ..

[46] Han E., Miller M.R., DeVeaux C., Jun H., Nowak K.L., Hancock J.T., Ram N., Bailenson J.N. (2023). People, places, and time: A large-scale, longitudinal study of transformed avatars and environmental context in group interaction in the metaverse. J. Comput.-Mediat. Commun..

[47] Gumusgul O., Acet M., Senturk A., Akin S., Isik U., Gumusgul C. (2025). The effect of exercise in virtual environment on psychological well-being and motivation for recreation participation. Curr. Psychol..

[48] Drazich B.F., Anokye D., Zhu S., Teleb J., Galik E., Colloca L., Resnick B. (2023). Motivating older adults through immersive virtual exercise (MOTIVE): A randomized pilot study. Geriatr. Nurs..

[49] World Health Organization (2002). Active Ageing: A Policy Framework.

[50] Takano M., Yokotani K., Kato T., Abe N., Taka F. Avatar Communication Provides More Efficient Online Social Support Than Text Communication. Proceedings of the International AAAI Conference on Web and Social Media.

[51] Elrod C.S., Pappa S.T., Heyn P.C., Wong R.A. (2023). Using an academic-community partnership model to deliver evidence-based falls prevention programs in a metropolitan setting: A community case study. Front. Public Health.

[52] Hill A.-M., Hoffmann T., Haines T.P. (2013). Circumstances of falls and falls-related injuries in a cohort of older patients following hospital discharge. Clin. Interv. Aging.

[53] Boright L.E., Arena S.K., Wilson C.M., McCloy L., Boright L. (2022). The effect of individualized fall prevention programs on community-dwelling older adults: A scoping review. Cureus.

[54] Ueda T., Higuchi Y., Hattori G., Nomura H., Yamanaka G., Hosaka A., Sakuma M., Fukuda T., Fukumoto T., Nemoto T. (2022). Effectiveness of a tailored fall-prevention program for discharged older patients: A multicenter, preliminary, randomized controlled trial. Int. J. Environ. Res. Public Health.

[55] Gong G., Na D. (2021). Current Status and Prospects for Commercialization of Wearable Robots for Gait Rehabilitation. Korea Robot. Soc. Rev..

[56] Sangyong S., Myeongul J., Kenny K.K. (2022). Impact of Virtual Avatar’s Appearance and Motion Synchrony on Sense of Embodiment: A Pilot Study with 3D Scanning Avatar Technology. J. Korea Comput. Graph. Soc..

[57] Koo D.-H. (2022). Future healthcare technologies for seniors and people with disabilities. Sports Sci..

[58] Goh D.H.-L., Razikin K. Is gamification effective in motivating exercise?. Proceedings of the Human-Computer Interaction: Interaction Technologies: 17th International Conference, HCI International 2015.

[59] Sailer M., Hense J.U., Mayr S.K., Mandl H. (2017). How gamification motivates: An experimental study of the effects of specific game design elements on psychological need satisfaction. Comput. Hum. Behav..

[60] Byeon H. (2024). Effects of Metaverse-Based Indoor Cycling Gamification on Physical and Cognitive Functions in Elderly Individuals with Mild Cognitive Impairment Using Bayesian Network Modeling. Nanotechnol. Percept..

[61] Strohacker K., Galarraga O., Williams D.M. (2014). The impact of incentives on exercise behavior: A systematic review of randomized controlled trials. Ann. Behav. Med..

[62] Mitchell M.S., Goodman J.M., Alter D.A., John L.K., Oh P.I., Pakosh M.T., Faulkner G.E. (2013). Financial incentives for exercise adherence in adults: Systematic review and meta-analysis. Am. J. Prev. Med..

[63] Kim J.-S., Cho J.-H. (2023). Development of the Metaverse of Customized Leisure Activity Support System for the Elderly. J. Korean Inst. Intell. Syst..

[64] World Health Organization (2019). WHO Guideline: Recommendations on Digital Interventions for Health System Strengthening.

[65] Proctor E., Silmere H., Raghavan R., Hovmand P., Aarons G., Bunger A., Griffey R., Hensley M. (2011). Outcomes for implementation research: Conceptual distinctions, measurement challenges, and research agenda. Adm. Policy Ment. Health.

[66] Agarwal S., LeFevre A.E., Lee J., L’engle K., Mehl G., Sinha C., Labrique A. (2016). Guidelines for reporting of health interventions using mobile phones: Mobile health (mHealth) evidence reporting and assessment (mERA) checklist. BMJ.

